# Constructing Cellulose Diacetate Aerogels with Pearl-Necklace-like Skeleton Networking Structure

**DOI:** 10.3390/gels7040210

**Published:** 2021-11-13

**Authors:** Shixian Xiong, Yangbiao Hu, Sizhao Zhang, Yunyun Xiao, Zhengquan Li

**Affiliations:** 1 China-Australia International Institute for Mineral, Metallurgy and Materials, Jiangxi University of Science and Technology, Ganzhou 341000, China; s.xiong@jxust.edu.cn (S.X.); biaoyanghu@163.com (Y.H.); zhengquan.li@jxust.edu.cn (Z.L.); 2 Jiangxi Provincial Key Laboratory for Simulation and Modelling of Particulate Systems, Jiangxi University of Science and Technology, Nanchang 330013, China

**Keywords:** cellulose diacetate, aerogels, freezing drying, pearl-necklace-like skeletons

## Abstract

Cellulose and its derivative aerogels have attracted much attention due to their renewable and biodegradable properties. However, the significant shrinkage in the supercritical drying process causes the relatively high thermal conductivity and low mechanical property of cellulose and its derivatives aerogels. Considering the pearl-necklace-like skeleton network of silica aerogels, which can improve thermal insulation property and mechanical property. Herein, we propose a new strategy for fabricating cellulose diacetate aerogels (CDAAs) with pearl-necklace-like skeletons by using tert-butanol (TBA) as exchange solvent after experiencing the freezing-drying course. CDAAs obtained have the low density of 0.09 g cm^−3^, the nanopore size in the range of 10–40 nm, the low thermal conductivity of 0.024 W m^−1^ K^−1^ at ambient conditions, and the excellent mechanical properties (0.18 MPa at 3% strain, 0.38 MPa at 5% strain). Ultimately, CDAAs with moderate mechanical property paralleled to cellulose-derived aerogels obtained from supercritical drying process are produced, only simultaneously owning the radial shrinkage of 6.2%. The facile method for fabricating CDAAs could provide a new reference for constructing cellulose/cellulose-derived aerogels and other biomass aerogels.

## 1. Introduction 

Aerogel is a solid material with a nanoscale skeleton and nonporous network structures, such as silica aerogels [1], chitosan aerogels [2], polybenzoxazine aerogels [3], etc. Aerogel has the unique characteristics of low density [4], high specific surface area [5], high porosity [6,7], low thermal conductivity [8], excellent absorptive activity [9,10], etc. Owing to the versatile characteristics, aerogel has been widely studied and successfully applied in thermal insulation [11,12], catalyst carrier [13,14], air purification, and water treatment [15,16]. However, synthetic processes and large-scale production of aerogel materials and the increasing consumption of non-renewable resources negatively impact the environment [17]. Therefore, the awareness of protecting the reduction of unrenewable resources impels us to develop in a sustainable direction, so biomass is an essential potential substitute with a feasible and friendly solution to the above problems [18].

Cellulose and its derivatives attract much attention based on biodegradability, renewability, environment friendliness, and a wide range of sources. Cellulose and its derivative aerogels have advantages of being lightweight, relatively high specific surface area and porosity [19], biodegradability, and biocompatibility [20,21,22]. Thus, cellulose and its derivative aerogels have broad application prospects in thermal insulation [23], adsorption separation [24,25], biomedicine [26,27], and electricity [28,29]. However, there is obvious shrinkage in the stage from the gel obtained after the process of aging and exchange solvent (final gel) to aerogel in the supercritical drying for cellulose/cellulose-derived aerogels, which produces negative effects for the industrial application of cellulose and its derivatives aerogels.

There is no or extremely low shrinkage from the final gel to aerogel on obtained cellulose and its derivatives aerogels via freezing drying course [30], but the corresponding mechanical property is poor [31]. Therefore, maintaining the relatively excellent mechanical property and superior thermal insulating property under no or extremely low shrinkage is challengeable. Schestakow [32] had studied the effects of exchange solvents such as water, ethanol, and acetone on the specific surface area, microstructure, and the mechanical property of cellulose aerogels. Results show that these solvents have an obvious impact on the specific surface area and mechanical performance of cellulose and its derivatives aerogels, but have little effect on the skeleton structure, the cellulose aerogels still show a fine fiber-like skeleton structure. Hence, considering the pearl-necklace-like skeleton network of silica aerogels, which can improve the thermal insulation property and mechanical property. We put forward to a novel solution for fabricating cellulose diacetate (CDA) aerogels (CDAAs) with pearl-necklace-like skeletons by tert-butanol (TBA) as exchange solvent after the freezing-drying treatment.

In this work, CDAAs with pearl-necklace-like skeletons structure and nano-porous networks were prepared with TBA as exchange solvent to enhance the strength of gel, subsequently using freezing-drying method to maintain extremely low shrinkage of obtained aerogels in the drying process. Shrinkage from the final gel to aerogel and density of CDAAs were discussed. The chemical structure was performed by Fourier transform infrared (FTIR) and X-ray photoelectron spectrometer (XPS). Morphology and structural parameters of CDAAs were acquired by Field-emission scanning electron microscope (FESEM) and nitrogen adsorption-desorption. The thermal insulation and mechanical property of CDAAs were studied through thermal conductivities and stress-strain curves. The formation of pearl-necklace-like skeletons in exchange solvent was also discussed preliminarily.

## 2. Results and Discussion

### 2.1. Shrinkage and Density of CDAAs

**Figure 1:**
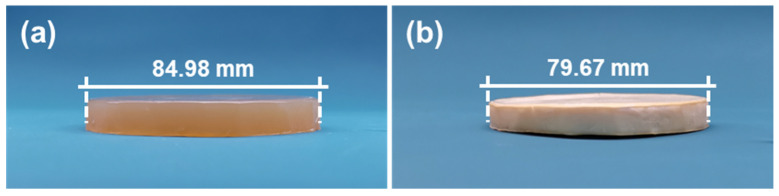
CDA final gel (**a**) and CDAAs (**b**).

CDA final gel and CDAAs are shown in Figure 1. There is low shrinkage from the final gel to aerogel, and the radial shrinkage is only 6.2%. As shown in Figure 1b, CDAAs are milky white, opaque, and lightweight (low density of ~0.09 g cm^−3^) due to the low shrinkage. The results are related to the degree of crosslinking [33]. There is no shrinkage in the aging and solvent exchange process, which is relative to the increscent degree of crosslinking, and low shrinkage in the freezing drying process is relative to the decreasing degree of crosslinking. Thus, the freezing drying means makes for inhibiting the shrinkage produced in the supercritical drying process for CDAAs.

### 2.2. Morphology and Structure of CDAAs

Through the initial research of the distinction between the surface morphology and the cross part of CDAAs, it was found that the surface portion is very compact and glossy due to the increase of reactant content caused by aging and rapid depletion of solvents in the incipient drying process, and only the cross part shows a characteristic nanonetwork structure. Therefore, to obtain the best perspective of CDAAs network structure, the cross-sectional part of CDAAs was observed by FESEM.

**Figure 2:**
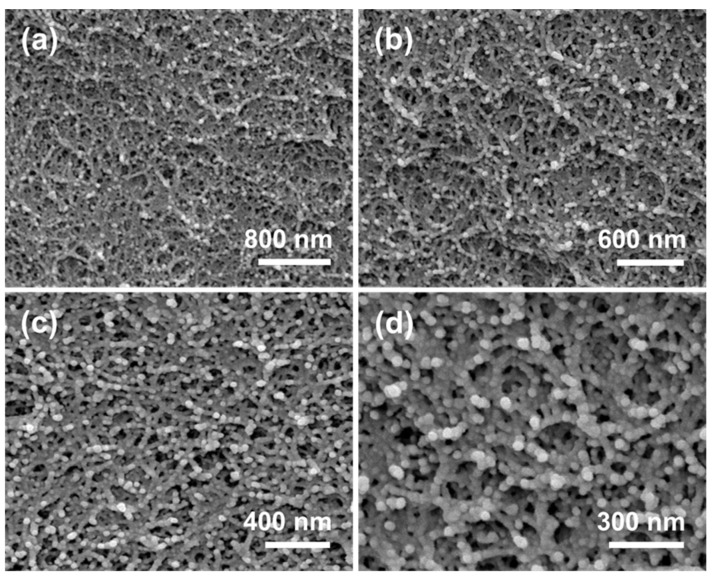
(**a–d**) FESEM images of CDAAs.

FESEM images of CDAAs with different resolutions are shown in Figure 2. CDAAs exhibit a 3D network with pearl-necklace-like skeletons, owing to the treatment of TBA, CDA gel particles formed in the initial nucleation stage continue to grow in the subsequent aging and solvent replacement stage, and finally form aerogel particles with a size of approximately 30 nm, which contributed to the reinforcement of CDAAs network. On the other hand, CDAAs have relatively dense and uniform pore sizes, and the pore sizes are about 10–50 nm. Robust pearl-necklace-like skeletons of CDAAs could make a significant contribution to the superior thermally-insulating property.

We also compare the microstructure of CDAAs and typical cellulose aerogels (CDBAs) by using acetone as the solvent [34]. Interestingly, the skeleton structure and the pore structure are observed by FESEM for CDAAs produced using TBA as exchange solvent and CDBAs are quite dissimilar. CDBAs exhibited a 3D network with fiber-like skeletons, and the pore size is relatively large, while CDAAs showed pearl-necklace-like skeletons and relatively small uniform pore size. The difference in microstructure between the two types of aerogels might be due to the impact of different exchange solvent on the skeleton, and pore size in cellulose and its derivatives aerogels. However, the real mechanism remains to be further explored.

**Figure 3:**
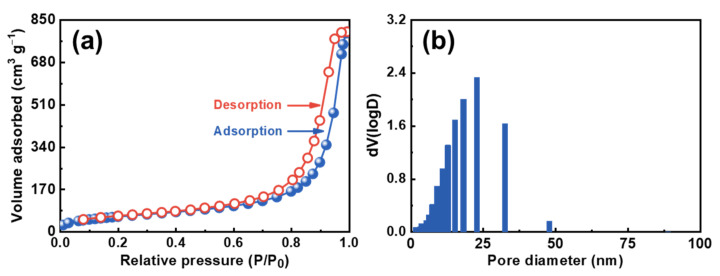
N_2_ adsorption-desorption isotherms (**a**) and pore size distribution (**b**) of CDAAs.

The specific surface area of CDAAs was analyzed through the BET method. The characteristic IUPAC-IV nitrogen adsorption-desorption isotherms of CDAAs within a hysteresis loop under the high relative pressure (P/P_0_ is above 1) is observed in Figure 3a, indicating the beingness of mesopores in CDAAs. The BET surface area is 223.4 m^2^ g^−1^, and the pore volume is 1.2 cm^3^ g^−1^ of CDAAs. The pore size distribution based on the BJH means is displayed in Figure 3b, and the pore size fluctuates from 10 to 40 nm, concentrated at about 23 nm, further indicating that the pore size of CDAAs is within the mesoporous reach of 2 to 50 nm, according to the IUPAC convention. Furthermore, the pore size distribution range measured by nitrogen adsorption-desorption is basically consistent with the measure by FESEM of 10–50 nm.

### 2.3. Chemical Crosslinking Evidence

**Figure 4:**
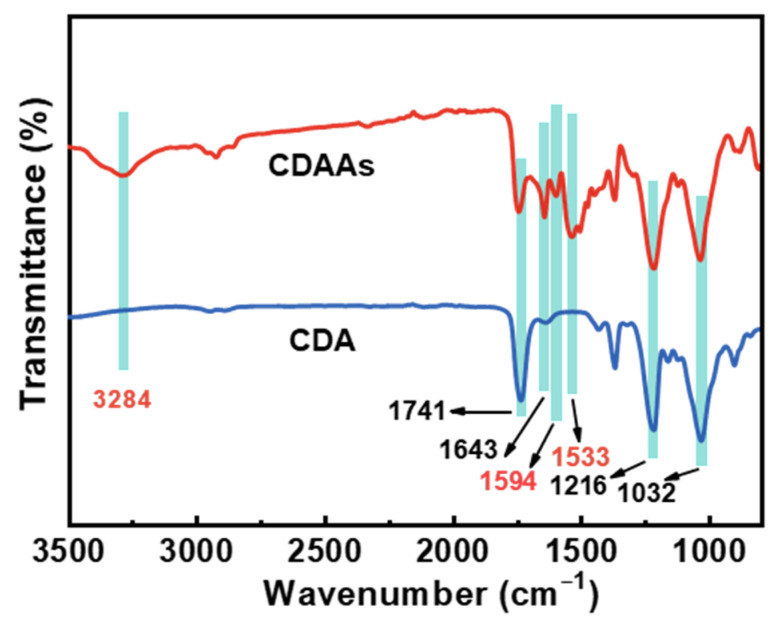
FTIR spectra of CDAAs and CDA.

**Figure 5:**
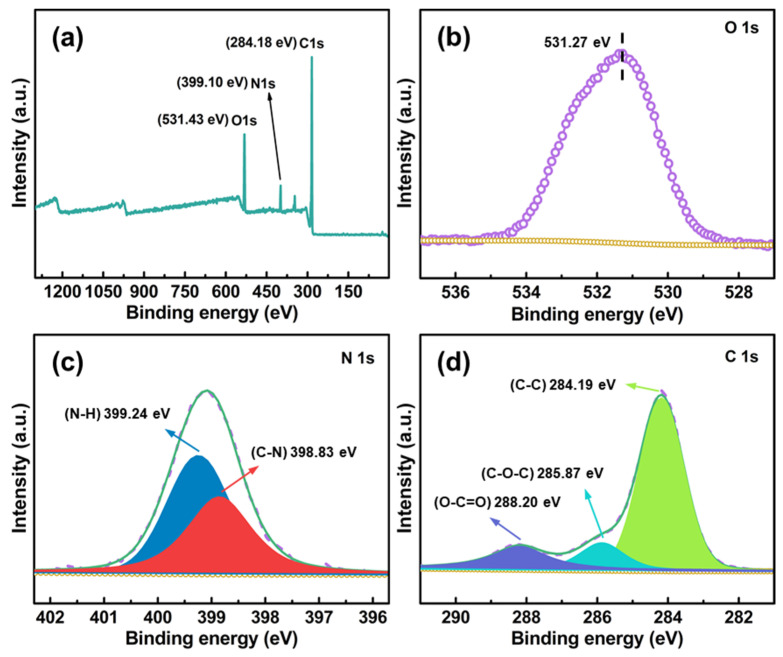
XPS spectra of CDAAs. Full survey (**a**), O 1s (**b**), N 1s (**c**), and C 1s (**d**).

FTIR spectra and XPS survey illustrate the proof of cross-linking reactions on CDAAs are described in Figure 4 and Figure 5. Figure 4 displays the FTIR spectrum of CDA and CDAAs, and representative characteristic absorption peaks are analyzed in detail. The absorption peak at 3284 cm^−^^1^ belongs to the stretching vibration of N-H [35], the peaks at 1741 and 1643 cm^−1^ represent the stretching and bending vibration of C=O [36], 1594 cm^−1^ is attributed to stretching vibration of C=C in benzene ring, 1533 cm^−1^ is for the stretching vibration of C-N and bending vibration of N-H, and 1216 cm^−1^ for the stretching vibration of C-O, respectively. Wherein absorption peaks of 1741 and 1216 cm^−1^ exist in both CDA and CDAAs, indicating that there are ester groups in the two materials, which corresponds to their molecular structures. The differences are the characteristic peaks of 3284 cm^−1^ (N-H), 1594 cm^−1^ (C=C), and 1533 cm^−1^ (C-N, N-H) that obviously appear in CDAAs, while no absorbed peaks in CDA, implying that TDI is successfully in cross-linking with CDA. It is worth noting that the characteristic peak with -N=C=O (~2210 cm^−1^) is not discovered in CDAAs, which indicates a secondary reaction between -N=C=O and ammonia ester bonds (-O-CONH-) occurred to form the allophanate structure (-HNCON-).

XPS further verifies the cross-linking of TDI with CDA, as shown in Figure 5, CDAAs mainly contain three elements including O, C, and N. The characteristic peaks at 531.43, 399.10, and 284.18 eV correspond to O 1s, N 1s, and C 1s, respectively. Wherein, in the spectra of N 1s (Figure 5c), the peak binding energies (BEs) located at 398.83 and 399.24 eV are attributed to C-N and N-H, respectively. Meanwhile, the BE peaks at located at 284.19 eV correspond to C-C, 285.87 eV is for C-O-C, and 288.20 eV belongs to O-C=O in C 1 s spectra (Figure 5d). Photoelectron absorption peaks in C 1s and N 1s further confirm cross-linking of the TDI and CDA, which is also consistent with the FTIR spectra of CDAAs.

**Figure 6:**
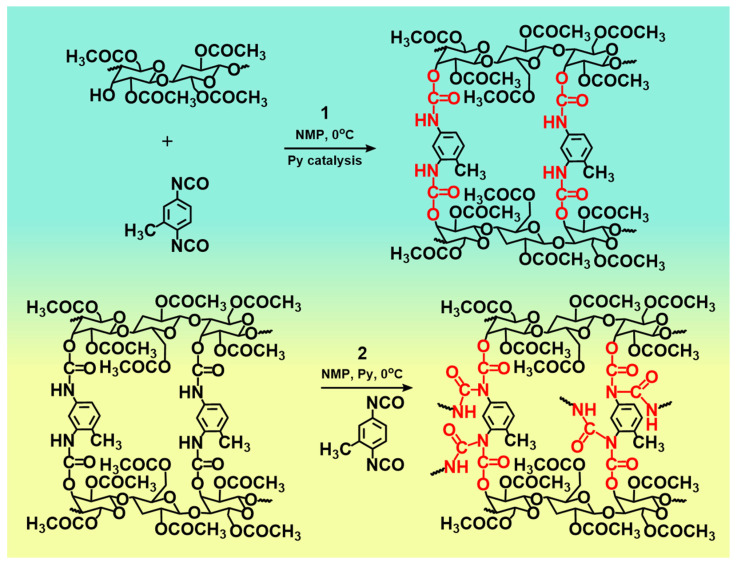
Reaction processes of CDAAs.

Chemical crosslinking plays an important role in the construction of CDAAs network. Figure 6 shows the synthesis route of CDAAs, this process mainly consists of two steps. Firstly, under the catalysis of pyridine, the hydroxyl group (-OH) upon the main molecular chain of CDA reacts with the -N=C=O groups on the cross-linking agent (TDI) through the hydrogen transfer reaction to form ammonia ester bonds (-O-CONH-). Secondly, the residual TDI in the system will further undergo hydrogen transfer reaction with the formed -O-CONH- bonds to obtain the allophanate structure [37] (-HNCON-) and further promote the cross-linking of CDA. Otherwise, there are the strong hydrogen bonds among -OH on CDA molecular chain to occur strong hydrogen bond interaction. Thus, the chemical cross-linking and the hydrogen bond interaction between CDA and TDI promoted the formation of the 3D network structure of CDDAs.

### 2.4. Thermal Conductivities of CDAAs

**Figure 7:**
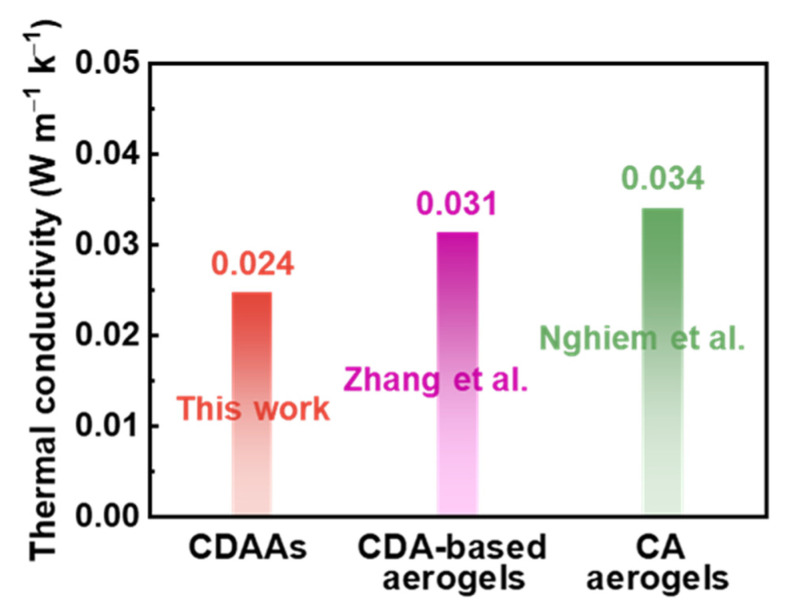
Thermal conductivities of CDAAs, CDA-based aerogels [40], and CA aerogels [41].

Thermal conductivities of CDAAs were tested via transient hot-wire theory under ambient temperature, which is 0.024 W m^−1^ K^−1^. Thermal conductivities of aerogels under ambient atmosphere could be briefly divided into two sections under neglecting the radiation conduction and the coupling with solid conduction at room temperature conditions: the solid thermal conductivity and the gaseous thermal conductivity [38]. Essentially, the solid thermal conduction of nanostructured materials is principally via touching solid skeletons in network structure, possess connections with the feature dimensions of the solid matrix touched; the gaseous thermal conduction is via gas convection, could be effectively impacted through pore size [39]. CDAAs obtained 3D network with pearl-necklace-like skeletons have relatively dense and uniform pore size, and the pore size is about 10–50 nm. Thus, gaseous thermal conduction has been reduced in a large part, and the low density (0.09 g cm^−3^) ensures the low solid thermal conductivity. The reported CDA-based aerogels [40] and CA aerogels [41] possessed thermal conductivities of 0.031 and 0.034 W m^−1^ K^−1^, respectively, which obviously are below that of CDAAs (0.024 W m^−1^ K^−1)^ in Figure 7. Therefore, TBA as exchange solvent has a positive effect in adjusting pore size and skeleton structure, which reduced thermal conductivities of CDAAs.

### 2.5. Compression Properties of CDAAs

**Figure 8:**
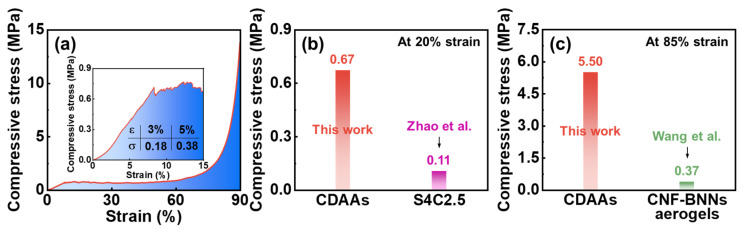
Compressive stress-strain curves of CDAAs (**a**); The compressive strength of CDAAs and S4C2.5 [42] at 20% strain (**b**); The compressive strength of CDAAs and CNF-BNNs [43] aerogels at 85% strain (**c**).

The compressive stress-strain curves of CDAAs in Figure 8a, the corresponding compressive strength is 0.18 and 0.38 MPa at 3 and 5% strain, respectively, indicating excellent mechanical properties of CDAAs. In previous studies, the cellulose aerogels of S4C2.5 [42] is only 0.11 MPa at 20% strain, and CNF-BNNs [43] is only 0.37 MPa at 85% strain, even below that of CDAAs at 5% strain, far below the compressive strength of CDAAs at 20% (0.67 MPa) and 85% strain (5.50 MPa) as shown in Figure 8b,c. Furthermore, the compressive strength of cellulose and its derivatives aerogels via the freezing-drying method usually is very low. However, CDAAs possess an excellent mechanical strength, which might be that the TBA as exchange solvent plays a crucial role in enhancing the skeleton structure of CDAAs. Meanwhile, the freezing-drying method also avoids the high pressure caused by the supercritical drying method, which can lay the foundation for realizing large-scale production and speeding up the application process of CDAAs.

### 2.6. Thermal Stability

**Figure 9:**
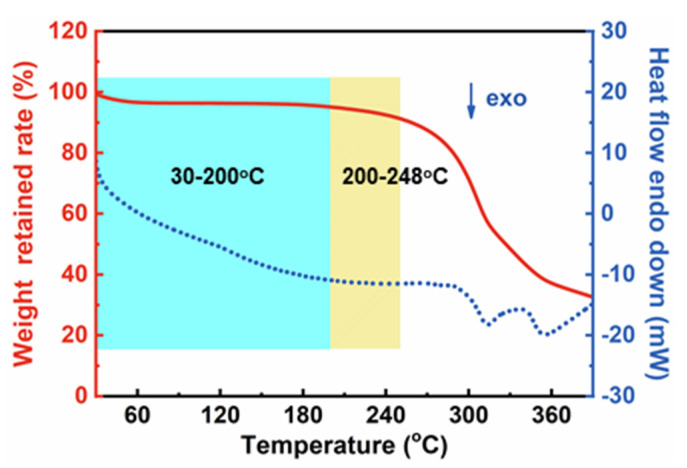
TG and DSC curves of CDAAs in nitrogen from 30 to 400 °C.

The mass evolutions of CDAAs were tested by the TG method under N_2_ atmosphere from 30 to 400 °C. The TG and DSC curves are presented in Figure 9**.** The weight loss processes of CDAAs are mainly divided into two stages: 30–248 °C and 248–400 °C. Below 200 °C, the mass loss is less than 5 wt%, and the mass loss of CDAAs reaches 10 wt% on the temperature reaches 248 °C. Weight loss in the range of 30–248 °C is mainly owing to moisture loss from the surface, absorbed by the internal porous structure, and bonded with hydrogen, as well as other small molecules in CDAAs. When the temperature is higher than 248 °C, the weight loss of CDAAs is gradually accelerated, which might be due to the breaking of the bonds of C-N, C-C, and Ar-C in CDAAs. With the further increase of the temperature, CDAAs continue to carbonize and the final mass retention rate is 32.65 wt% at 400 °C.

## 3. Conclusions

In conclusion, CDAAs with pearl-necklace-like skeletons were prepared successfully under the chemical crosslinking and the hydrogen bonding interaction by freezing drying. TBA as exchange solvent made a great contribution to the formation of CDAAs with the pearl-necklace-like structure. CDAAs showed a 3D nano-porous network structure with a pore size of 10–40 nm, low density of 0.09 g cm ^3^, low shrinkage (6.2%), and specific surface area (223.4 m^2^ g^−1^). Meanwhile, CDAAs possessed excellent mechanical properties (0.18 MPa at 3% strain, 0.38 MPa at 5% strain), low thermal conductivity (0.024 W m^−1^ K^−1^) at ambient environment, and superior thermal stability, and the weight loss was less than 5 wt% at 200 °C. This facile method for the preparation of CDAAs realizes the excellent mechanical property and superior thermal insulating property under no or extremely low shrinkage, and would potentially promote the industrial production and practical application of cellulose and its derivatives aerogels, and may provide a basis for the preparation of biomass aerogels.

## 4. Materials and Methods

### 4.1. Materials

Cellulose diacetate (CDA, 39.8 wt% acetyl content), 2, 4-toluene diisocyanate (TDI), pyridine (Py, AR), tert-butanol (TBA, AR), and n-methyl pyrrolidone (NMP, AR) were obtained from Shanghai Aladdin Biochemical Technology Co., Ltd. (Shanghai, China). Deionized and doubly distilled water was used directly. All chemicals were directly used without any additional purification. 

### 4.2. Preparation of CDAAs

**Figure 10:**
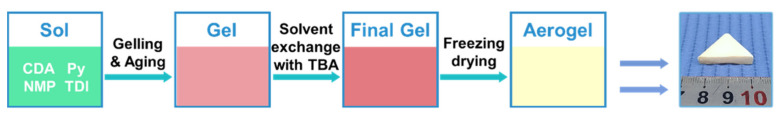
Illustration of the fabrication process of CDAAs.

The synthetical pathway for CDAAs was displayed in Figure 10. CDA was dissolved in NMP to a homogeneous solution after stirring for 2 h. Then, the pyridine was dropped into the above solution to stir for 1.5 h, and the TDI was added in the mixed solution to stir for 30 min to obtain the CDA sol. The weight ratio of CDA and TDI was designed to 1.22:1. The sol was quickly moved into the cylindrical mold (radius: 100 mm, height: 20 mm), and sealed in an oven at 60 °C to form the CDA gel. Whereafter, the gel prepared was covered by NMP at 60 °C for aging for 2 days. Then, the aged gel was soaked in a certain proportion of TBA in 75 °C to exchange NMP, and exchanging 4–6 times every day for 4–5 days. Eventually, CDAAs were acquired with freezing drying at 1 Pa and −85 °C. Fabrication processes of CDAAs regarding gel, and aerogel photo can be seen in Figure 1.

### 4.3. Characterizations

The bulk density ($\rho_{c}$) with CDAAs was accounted via dividing the mass ($m_{c}$) of CDAAs by the volume ($V_{c}$).

(1) $$\;\;\rho_{c}=\frac{m_{c}}{V_{c}}$$

The linear shrinkage (S) of CDAAs was calculated using the equation as follows. The diameter (D_0_) with final gel and the diameter (D) with CDAAs were recorded using a Vernier caliper

(2) $$S=\frac{D_{0}-D}{D_{0}}\times 100\%$$

The morphology of CDAAs was obtained via Field-emission scanning electron microscope (FESEM) (S4800, Hitachi Limited, Tokyo, Japan) wsithin metal spraying, and the accelerating voltage was set to 3 kV and the working distance was 5.1 mm. The nitrogen adsorption-desorption test was executed with the Autosorb IQ analyzer (ASAP 2020 HD88, Mike instruments, Atlanta, Georgia, USA). The sample was degassed before testing, and the degassing temperature and time were 90 °C and 12 h, respectively. The specific surface area (S_BET_) was detected by the Brunauer-Emmett-Teller (BET) means, and the total pore volume (V_p_) and the diameter of pore (D_p_) were detected with Barrett-Joyner-Halenda (BJH) analysis from the desorption branch.

The chemical structure of CDAAs was detected using Fourier transform infrared (FTIR) spectrophotometer (Nicolet iS5, Thermo Fisher Scientific, Waltham, MA, USA) (in the area of 400–4000 cm, the ratio of the weight of CDAAs to KBr is 1:99), and the X-ray photoelectron spectrometer (XPS) employed Mg Kα radiation (Tescalab-250Xi, Thermo Fisher Scientific, Waltham, MA, USA). Thermal stabilities of CDAAs were mensurated with thermogravimetry (TG) and differential scanning calorimetry (DSC) (Sta 449F3, Netzsch instruments Manufacturing Co., Ltd., Selb, Germany), and the heating rate is 5 °C min^−1^ in N_2_ atmosphere at 30–400 °C.

Before the test for mechanical property, the dense surface of the sample was grinded off and the internal part was used for measurement and the compression strength was assessed by general test machine (XBD4204, Sansi Yongheng Technology Co., Ltd., Zhejiang, China) on a load cell of 20 KN. The compression rate was 0.5 mm min^−1^. Thermal conductivities of specimens were tested via the thermal conductivity instrument (TC3000E, Xi’an Xiaxi Electronic Technology Co., Ltd., Xi’an, China) with transient hot-wire theory at 25 °C.

## Data Availability

Data are available from the authors. Samples of the compounds are available from the authors.
